# Relative Validity of Three Food Frequency Questionnaires for Assessing Dietary Intakes of Guatemalan Schoolchildren

**DOI:** 10.1371/journal.pone.0139125

**Published:** 2015-10-14

**Authors:** Jessica Marcinkevage, Ana-Lucia Mayén, Clara Zuleta, Ann M. DiGirolamo, Aryeh D. Stein, Manuel Ramirez-Zea

**Affiliations:** 1 Laney Graduate School, Graduate Division of Biological and Biomedical Sciences, Emory University, Atlanta, Georgia, United States of America; 2 INCAP Research Center for the Prevention of Chronic Diseases (CIIPEC), Institute of Nutrition of Central America and Panama, Guatemala; 3 Hubert Department of Global Health, Rollins School of Public Health, Emory University, Atlanta, Georgia, United States of America; 4 Andrew Young School of Policy Studies, Georgia State University, Atlanta, Georgia, United States of America; University of São Paulo, BRAZIL

## Abstract

**Objective:**

To determine the relative validity of three food frequency questionnaires (FFQs) compared with results from 24-hour dietary recalls for measuring dietary intakes in Guatemalan schoolchildren.

**Design:**

A cross-sectional study of primary caregivers (mothers or grandmothers) of 6–11 year-old children. Caregivers completed one of three constructed FFQs to measure the child’s dietary consumption in the last week: FFQ1 did not incorporate portion sizes; FFQ2 provided portion sizes; and FFQ3 incorporated pictures of median portion sizes. During the same week, each caregiver also completed three 24-hour dietary recalls. Results from the FFQ were compared with corresponding results from the 24-hour dietary recalls.

**Setting:**

Santa Catarina Pinula, peri-urban Guatemala City.

**Subjects:**

Caregivers (n = 145) of 6–11 year-old children: 46 completed FFQ1, 49 completed FFQ2, and 50 completed FFQ3.

**Results:**

The mean values for all nutrients obtained from the 24-hour dietary recall were lower than for those obtained from the FFQs, excluding folic acid in FFQ3, cholesterol and zinc in FFQ2, and cholesterol, folic acid, magnesium, potassium, sodium, and zinc in FFQ1. Energy-adjusted Pearson correlation coefficients ranged from 0.07 (protein) to 0.54 (cholesterol) for FFQ1 and from 0.05 to 0.74 for FFQ2 and FFQ3. Agreement by both methods (FFQ and 24-hour dietary recalls) of classifying children into the same or adjacent quartiles of energy-adjusted nutrient consumption ranged from 62.0% for cholesterol to 95.9% for vitamin B12 across all three FFQs.

**Conclusions:**

Our FFQs had moderate to good relative validity in measuring energy and nutrient intakes for 6–11 year-old Guatemalan children. More evidence is needed to evaluate their reproducibility and applicability in similar populations.

## Introduction

A significant increase in non-communicable diseases (NCDs) has been observed in lower- and middle-income countries as societies face both a demographic and epidemiologic transition [1, 2]. The nutrition transition subsequently occurring in these societies is characterized by a shift in dietary consumption patterns toward more energy dense diets, coupled with a reduction in physical activity [3]. These dietary patterns—characterized by high amounts of sugar, salt and saturated fats—are considered a main risk factor of NCDs [4], and usually appear first in high socioeconomic status (SES) individuals [5], leading to increased prevalence of overweight and obesity. Meanwhile, nutritional deficiencies persist in their disadvantaged counterparts, creating a double burden of malnutrition [6].

Different approaches exist for estimating dietary consumption patterns and nutrient intakes [7, 8], though none has proven to be ideal [8]. The food frequency questionnaire (FFQ) has been used to estimate an individual’s usual food intake over a defined period of time [9]. Though the FFQ may not perform as well as objective measures of dietary intake (e.g., doubly- labeled water) [10], it has been widely accepted for its use in epidemiological studies when compared with other methods of assessing dietary intake due to its relative low burden to participants and research staff, low cost, and high validity for measuring a multitude of nutrients [11].

Children have special dietary needs and requirements [12]; however the assessment of their dietary patterns is difficult due to reporting errors and dependence on surrogate respondents [7]. FFQs have been commonly used for measuring dietary intakes in children [13–16]. However, a FFQ developed for one population may not be adequate for use in another due to differences in dietary patterns and food preparations between populations [17].

Previous studies have utilized FFQs for assessing dietary intakes in children via different methods of measuring both frequency of consumption and quantity of food consumed. Examples include FFQs with pictures of food items [18–22], household measures for portion sizes [23, 24], or no portion size provided at all (rather, employing a reference to a median portion size within the population) [25–27]. There are advantages to using these different methods of measurement, including diminishing the complexity and response burden associated with the FFQ [28]. However, no studies exist evaluating the differences between the three types of FFQs when used in the same population, nor the most suitable of the three to be used for a specific population.

Guatemala is a lower middle-income country where evidence of the nutrition transition has been described (29). A previous study evidenced an excess of body weight in high socioeconomic status (SES) school-age Guatemalan children, compared to their low SES counterparts (17.7% versus 10.5%) [29]. This is occurring concomitantly with observed micronutrient deficiencies, specifically vitamin B12 in school-aged children, and iron in adolescents [30]. As dietary patterns among children are changing, the proper assessment of nutrient intakes for this group could prove useful in contributing to the development of adequate interventions for the prevention of both NCDs and micronutrient deficiencies. Despite these observations, there are no validated tools for assessing dietary intakes in Guatemalan children. Thus, the aim of our study was to develop three FFQs and assess their relative validity for measuring nutrient intakes in Guatemalan children living in two low-income villages at the outskirts of Guatemala City, by comparing results obtained from the FFQs to those obtained from a series of 24-hour dietary recalls.

## Methods

### Study sample

We recruited participants for the study from two public schools in two separate villages of Santa Catarina Pinula, on the outskirts of Guatemala City, Guatemala. These villages have participated in studies from our organization, the Institute of Nutrition of Central America and Panama (INCAP), over the past several years. Participants were eligible if they were caregivers of a child 6–11 years-old who attended 1^st^-4^th^ grades at either of the schools, and knowledgeable of the child’s usual food intake. In total, we recruited 147 caregivers, all of whom were either the child’s mother or grandmother. We obtained informed written consent from the caregivers prior to their enrollment into the study. The study was approved by the Ethics Committee of Roosevelt Hospital in Guatemala City, Guatemala.

### Study design

We conducted a cross-sectional analysis of children’s dietary intakes via three unique FFQs. Of the 147 caregivers recruited (69 of whom were caregivers to girls) two did not provide complete data on their child’s intakes and were not included in the analysis, resulting in a final sample size of 145. These women were interviewed one-on-one in-person by trained personnel, based on the protocol developed for this study. Caregivers were divided into three groups and assigned to answer one of three different types of FFQs, as described below. Each caregiver also completed 3 interviewer-administered 24-hour dietary recalls over 3 non-consecutive days (Monday, Wednesday and Friday). We administered FFQs at the end of the same week, on Saturdays. The 24-hour dietary recalls served as the comparison method for the FFQs.

### FFQ development

As the model for our three study FFQs, we used the INCAP semi-quantitative FFQ, which was originally designed to assess dietary intakes in an adult Guatemalan population [31]. This model FFQ included a list of 52 food items and was validated by comparing results with those obtained from the means of three non-consecutive 24-hour dietary recalls. Pearson correlation coefficients showed a moderate level of agreement between the two methods, at 0.64 for crude energy, 0.63 for both fat and carbohydrates, and ranging from 0.40 to 0.52 for selected micronutrients (calcium, thiamin, riboflavin and niacin) [31].

From this model FFQ, we created three different FFQs to include a list of 108 food items grouped by 21 main food categories (beans, pasta and rice, potatoes, tortillas, breads, cereal beverage (*atol*), cereals, soups, vegetables, fruits, dairy, eggs, meats, fats, fast foods, regional meals, drinks, snacks, sweets, complementary ingredients and micronutrient supplements). We added food items not already included in the original 52-item FFQ based on preliminary research within the community. This included discussions with community members, to ascertain which local foods were more frequently consumed by school-age children in that setting. Items that were consumed with medium and high frequency (i.e., more than 10 times) during the past 7 days were added to the original FFQ for a final total of 108 items.

Although all three FFQs of our study utilized the same food item list and measured the frequency of consumption in the last seven days, they differed by their presentation of portion sizes and assumptions for food consumption in children. FFQ1 assumed all children normally ate the median portion size measured in the population. The questionnaire therefore did not contain any measurement of portion size served or consumed, and utilized the median portion size for determination of nutrient intakes (n = 46 for FFQ1) FFQ2 did not make such assumptions, and instead included a standardized measurement of pre-established standardized portion sizes (food portions using household measures, including spoons, bowls, plates and cups; n = 49 for FFQ2) as selected by the caregiver. FFQ3 made the same assumptions as FFQ1, but utilized the median portion size as depicted in a photograph of each food item (n = 50 for FFQ3). Caregivers were then able to report if the child ate more, less or the same amount as that depicted in the photograph.

### 24-hour dietary recalls

The procedure for administering the 24-hour dietary recalls has been described elsewhere [32]. We asked each respondent to recall the type and amount of any food or beverage consumed by the child during the previous day, in chronological order. Individual food recipes were also taken into account. During the interview, we weighed all foods reported using an OHAUS food scale. Reported foods were measured in the home of each participant if available. Otherwise they were weighed in stores, supermarkets, and fast food restaurants indicated by the caregivers. When necessary, conversion factors were employed to convert raw/uncooked grams to cooked grams [33]. Data quality control procedures included a nutritionist’s daily supervision of all the fieldwork, revision of each form and the coding of each food according to the Food Composition Table of INCAP [34]. All recipes that were reported in the 24-hour dietary recalls but did not appear at INCAP’s food composition table were standardized via interviews with groups of 10 caregivers for each recipe. During this process, women were asked specifically how they prepared each recipe; their responses were averaged to derive an average measurement for the recipe in question. We excluded extreme values of energy (< 550 kcal or > 4000 kcal/day). Only those children with 2 or more 24-hour dietary recalls were included in the final analysis.

### Measurement of median portion sizes

To design FFQ1 and to create the Food Photograph Atlas for FFQ3, we measured median portion sizes consumed in the general population for each food item. We recruited 40 caregivers of children aged 6–11 years old, attending our two selected public schools but not participating in the study. Using a 24-hour dietary recall method, we asked respondents to report on the usual intakes and portions served to their children. We calculated the median portion value (in grams) of each food item from 10 or more responses provided on the 24-hour dietary recalls. In cases where 10 responses were not available from the 24-hour dietary recalls, we supplemented our data by interviewing 15 more caregivers. The information from these caregivers in the community also confirmed if the food items included in the FFQs were commonly consumed in the community.

### Development of Food Photograph Atlas

Using the calculated median portion size of each food item, we created a food photograph atlas to assist respondents in the estimation of portion sizes while answering FFQ3. For its validation, we recruited 23 caregivers of 7–10 year-old children, who resided in Mixco, a village located at the outskirts of Guatemala City with similar socio-economic and environmental characteristics as the study villages. We photographed the median portion size for 108 food items, which were previously weighed. We presented each caregiver with the picture of the median portion size of the food item, as well as the weighed food item. We asked the caregiver if the portion size in the picture was the same as the food item presented. Additionally we asked if the portion size in the picture represented what her child usually consumed.

### Statistical analysis

We analyzed food items in the FFQs and 24-hour dietary recalls as continuous variables. We calculated nutrient intake by multiplying the frequency of consumption (times per day and number of days) by the portion weight per 7 days, to derive a quantity of g/day. This quantity (in g/day) was multiplied by the nutrient content per 100 g according to INCAP’s food composition table. For FFQ1, in which there were no portion sizes provided, the median portion size (g) was used to assess the intake of energy and nutrients. For FFQ2, the grams of each portion corresponded to the portion size identified on the questionnaire; for FFQ3, the grams of each portion corresponded to the median portion size (g) shown in the picture. However, for FFQ3, if caregivers reported their child had eaten less or more than the portion size pictured, we utilized the 25^th^ or 75^th^ percentile, respectively, instead of the median, as the portion consumed. This methodology has been used previously to define small (25^th^ percentile) or large (75^th^ percentile) portion sizes of dishes [35].

We excluded all subjects with extreme values of energy intakes (< 550 kcal or > 4000 kcal/day), as we did with the information obtained with the 24-hour dietary recalls. To improve normality of some nutrients, we log-transformed reported intakes. We computed descriptive statistics for each FFQ and 24-hour dietary recall group. We compared group mean energy and nutrient intakes estimated by the 24-hour dietary recalls with those estimates obtained from the FFQ, using the paired sample t-test for each individual FFQ type. We repeated these analyses after adjustment for energy intake, using the residual method described by Willett, et. al [9] and further assessed agreement between the two methods by producing Bland-Altman plots, graphing the difference between the two methods against the average of the two methods [36]. In doing so, Bland-Altman plots can highlight the direction of bias between methods, if it exists, and whether this bias is constant across varying levels of intake.

We used Pearson correlation coefficients to compare the unadjusted and energy-adjusted nutrient intakes obtained from each FFQ versus the mean of three 24-hour dietary recalls, and corrected for measurement error due to within-subject variability in the multiple 24-hour dietary recalls, according to the method presented by Willet, et. al [9]. We evaluated concordance between the two methods with cross-classification of intake and nutrient distribution into quartiles. For these analyses, the expectation is 25% for exact agreement between the two methods, 65% for agreement within one quartile, and 12.5% for gross misclassification [37]. We also assessed agreement by calculating a weighted kappa statistic, using weight = 1 for total agreement, weight = 0.66 if differed by 1 category, weight = 0.33 if differed by 2 categories, and weight = 0 if grossly misclassified. All data were entered using Microsoft Excel, and data analyses were completed using SAS version 9.2 (SAS Institute, Cary, NC, USA).

## Results

The mean age (SD) of children for whom caregivers were interviewed for FFQ1, FFQ2 and FFQ3 were 8.5 (1.2), 8.0 (1.0) and 8.2 (1.2) years, respectively. Unadjusted and energy-adjusted macro- and micronutrient intakes showed similar patterns of consumption when assessed by each of the three FFQs. However, the three FFQs generally overestimated daily nutrient intake (Tables 1–3). FFQ2 and FFQ3 significantly overestimated the mean energy intake compared to the corresponding 24-hour dietary recalls (p = 0.01 and p<0.001, respectively). Most intakes of macro- and micro- nutrients (unadjusted and energy-adjusted) assessed by FFQ2 and FFQ3 were significantly higher (p<0.05) than the corresponding value from the 24-hour dietary recalls, using student's paired t-tests.

**Table 1:** Mean (standard deviation [SD]) energy and nutrient intakes, and Pearson’s correlation coefficients comparing a food frequency questionnaire without portion sizes (FFQ1), with the average of three 24-hour dietary recalls in Guatemalan schoolchildren 6–11 years old.

	FFQ1 (n = 46)						
	24-hr recall mean	SD	FFQ mean	SD	r	r_adj_	r_adj,corr_
Energy (kcal)	1767.1	408.2	1816.1	705.1	0.06		
Carbohydrate (g)	277.3	70.6	288.5	120.0	-0.03	0.14	0.17
Fat (g)	51.0	17.5	52.0	20.7	0.26	0.13	0.18
Protein (g)	56.8	15.2	56.8	20.3	0.02	0.07	0.10
Iron (mg)	16.7	12.0	17.6	8.51	0.24^a^	0.29^a^	0.48^a^
Calcium (mg)	590.0	324.2	635.5	348.2	0.05	0.37	0.58
Niacin (mg)	14.1	5.0	15.1	6.4^a^	0.27^a^	0.35	0.83
Riboflavin (mg)	1.3	0.5	1.4	0.7^a^	0.37^a^	0.53	0.57
Thiamin (mg)	1.0	0.4	1.0	0.5^a^	0.15^a^	0.21^a^	0.30^a^
Pyridoxine (mg)	1.2	0.5	1.3	0.7^a^	0.32^a^	0.30	---
Cholesterol (mg)	236.1	121.7	191.1	79.0 ^a^ ^,^ *	0.43^a^	0.54^a^	---
Folic acid (μg)	172.2	116.6	162.8	107.2	0.23	0.20	0.32
Vitamin B12 (μg)	3.3	2.0	3.6	2.1^a^	0.44^a^	0.45	---
Zinc (mg)	5.5	2.1	4.9	2.0	0.21	0.33	0.59
Magnesium (mg)	133.2	43.7	120.5	56.6	0.14	0.24	0.82
Potassium (mg)	1513.1	533.4	1451.2	699.2	0.17	0.27	0.49
Sodium (mg)	1011.0	517.8	583.9	352.5^a^ ^,^ *	0.46^a^	0.37	0.52
Vitamin A (RE)	768.3	467.1	794.3	611.7^a^	0.39^a^	0.42^a^	0.69^a^
Vitamin C (mg)	57.6	38.8	74.6	63.4^a^	0.33^a^	0.40^a^	0.57^a^

FFQ1, Food frequency questionnaire without portion sizes.

Adj. = energy-adjusted nutrients used for the analysis; corr. = analysis corrected for measurement error; RE, retinol equivalents.

^a^ Log-transformed values were used for these analyses.

--- Calculated corrected correlation coefficient > 1.0.

* Denotes values statistically different (p<0.05) than mean values obtained from 24-hour dietary recalls, using Student’s t-test.

**Table 2:** Mean (standard deviation [SD]) energy and nutrient intakes, and Pearson’s correlation coefficients comparing a food frequency questionnaire with portion sizes (FFQ2), with the average of three 24-hour dietary recalls in Guatemalan schoolchildren 6–11 years old.

	FFQ2 (n = 49)						
	24-hr recall mean	SD	FFQ mean	SD	r	r_adj_	r_adj,corr_
Energy (kcal)	1721.3	403.7	2388.8	883.9*	0.46		
Carbohydrate (g)	273.2	62.8	455.8	201.2*	0.32	0.06	0.08
Fat (g)	48.7	15.7	91.3	36.4*	0.30	0.04	0.06
Protein (g)	53.1	15.3	84.1	34.8*	0.37	0.05	0.06
Iron (mg)	14.4	4.9	33.5	33.9^a^ ^,^ *	0.43^a^	0.64^a^	0.81^a^
Calcium (mg)	527.4	195.3	968.0	452.1*	0.36	0.24	0.28
Niacin (mg)	12.8	4.9	65.4	46.7*	0.11	0.15^a^	0.21^a^
Riboflavin (mg)	1.2	0.5	2.1	2.8*	0.65	0.74^a^	0.87^a^
Thiamin (mg)	0.9	0.4	1.8	2.2^a^ ^,^ *	0.51^a^	0.32^a^	0.42^a^
Pyridoxine (mg)	1.0	0.6	2.1	3.6*	0.66	0.72^a^	0.88^a^
Cholesterol (mg)	245.6	194.0	190.8	96.7^a^ ^,^ *	0.63^a^	0.17^a^	0.25^a^
Folic acid (μg)	168.6	112.3	351.2	491.7*	0.23	0.30	0.45
Vitamin B12 (μg)	2.8	3.1	4.3	10.1	0.65	0.66^a^	0.79^a^
Zinc (mg)	4.5	1.7	4.2	2.0	0.49	0.41	0.50
Magnesium (mg)	119.8	40.0	147.4	86.3*	0.34	0.27	0.45
Potassium (mg)	1282.8	440.4	1389.1	608.1	0.46	0.26	0.38
Sodium (mg)	971.1	502.3	1043.9	797.8	0.41	0.23^a^	0.34^a^
Vitamin A (RE)	655.3	288.5	732.6	672.4	0.51	0.35^a^	0.45^a^
Vitamin C (mg)	52.9	37.3	67.8	45.7^a^ ^,^ *	0.62^a^	0.67^a^	0.86^a^

FFQ2, Food frequency questionnaire with portion sizes. Adj. = energy-adjusted nutrients used for the analysis; corr. = analysis corrected for measurement error; RE, retinol equivalents.

^a^ Log-transformed values were used for these analyses.

--- Calculated corrected correlation coefficient > 1.0.

* Denotes values statistically different (p<0.05) than mean values obtained from 24-hour dietary recalls, using Student’s t-test.

**Table 3:** Mean (standard deviation [SD]) energy and nutrient intakes, and Pearson’s correlation coefficients comparing a food frequency questionnaire with photos (FFQ3), with the average of three 24-hour dietary recalls in Guatemalan schoolchildren 6–11 years old.

	FFQ3 (n = 50)						
	24-hr recall mean	SD	mean	SD	r	r_adj_	r_adj,corr_
Energy (kcal)	1566.0	493.0	1917.5	769.2*	0.40		
Carbohydrate (g)	258.7	83.8	289.1	103.2*	0.34	0.28	0.37
Fat (g)	41.9	19.0	61.1	34.0*	0.45	0.28	0.37
Protein (g)	47.7	14.6	62.3	27.8*	0.48	0.41	0.54
Iron (mg)	14.2	4.9	17.3	8.4*	0.39	0.09	0.37
Calcium (mg)	530.3	210.2	807.4	519.0^a^ ^,^ *	0.47^a^	0.17^a^	0.24^a^
Niacin (mg)	12.1	5.0	14.6	7.1*	0.49	0.28	0.71
Riboflavin (mg)	1.0	0.4	1.5	0.9*	0.64	0.39	0.63
Thiamin (mg)	0.9	0.3	1.2	0.5*	0.56	0.31	0.48
Pyridoxine (mg)	1.0	0.4	1.5	1.0*	0.54	0.48	---
Cholesterol (mg)	190.8	105.6	285.6	175.2*	0.29	0.18	0.30
Folic acid (μg)	155.2	87.4	122.3	134.3^a^ ^,^ *	0.18^a^	0.11^a^	0.23^a^
Vitamin B12 (μg)	2.5	3.1	3.7	3.0^a^ ^,^ *	0.66^a^	0.51^a^	0.83^a^
Zinc (mg)	4.3	1.7	5.8	3.5*	0.56	0.38	0.73
Magnesium (mg)	131.2	60.0	160.2	98.0^a^ ^,^ *	0.40^a^	0.35^a^	0.56^a^
Potassium (mg)	1363.5	567.1	1829.7	1130.7^a^ ^,^ *	0.44^a^	0.35^a^	0.86^a^
Sodium (mg)	831.8	442.5	1078.9	697.2^a^	0.07^a^	-0.09^a^	-0.13^a^
Vitamin A (RE)	667.2	716.4	801.7	646.1^a^	0.33^a^	0.30^a^	0.44^a^
Vitamin C (mg)	61.1	42.6	121.9	102.6^a^ ^,^ *	0.46^a^	0.42^a^	---

FFQ3, Food frequency questionnaire with pictures of median portion sizes. Adj. = energy-adjusted nutrients used for the analysis; corr. = analysis corrected for measurement error; RE, retinol equivalents.

^a^ Log-transformed values were used for these analyses.

--- Calculated corrected correlation coefficient > 1.0.

* Denotes values statistically different (p<0.05) than mean values obtained from 24-hour dietary recalls, using Student’s t-test.

**Fig 1:**
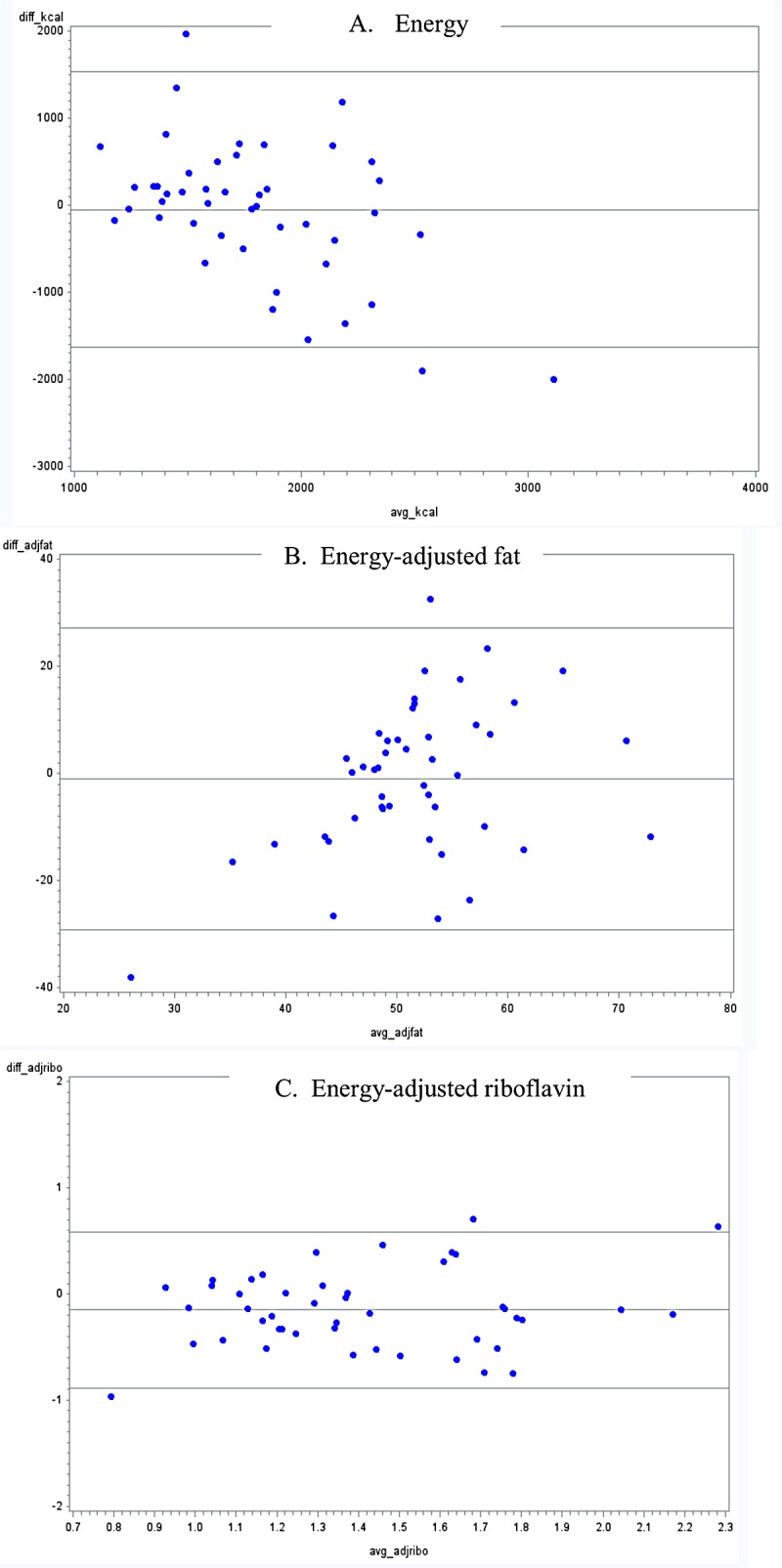
Bland-Altman plots for selected nutrients comparing a food frequency questionnaire without portion sizes (FFQ1), with the average of three 24-hour dietary recalls in Guatemalan schoolchildren 6–11 years old.

**Fig 2:**
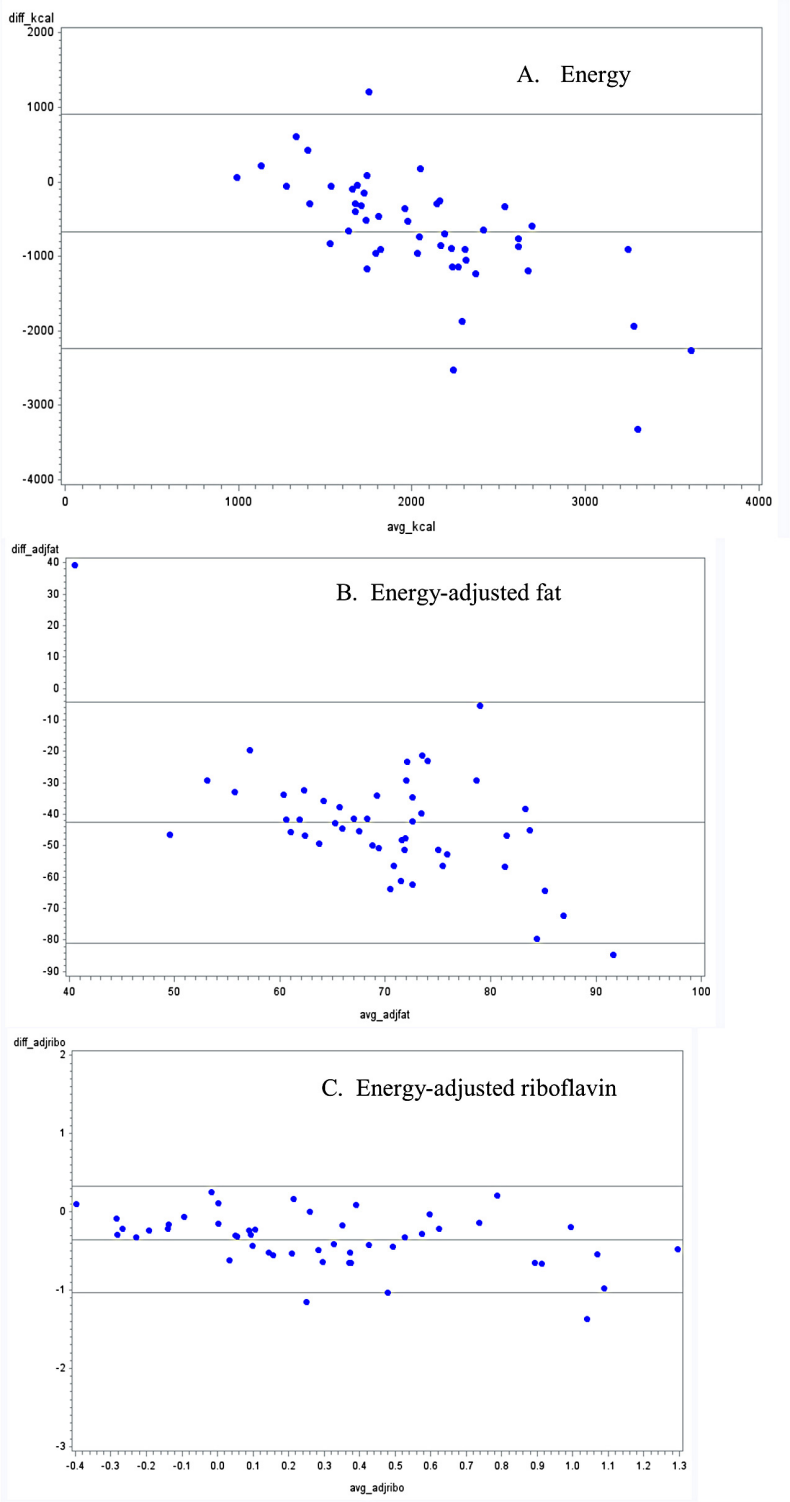
Bland-Altman plots for selected nutrients comparing a food frequency questionnaire with portion sizes (FFQ2), with the average of three 24-hour dietary recalls in Guatemalan schoolchildren 6–11 years old.

**Fig 3:**
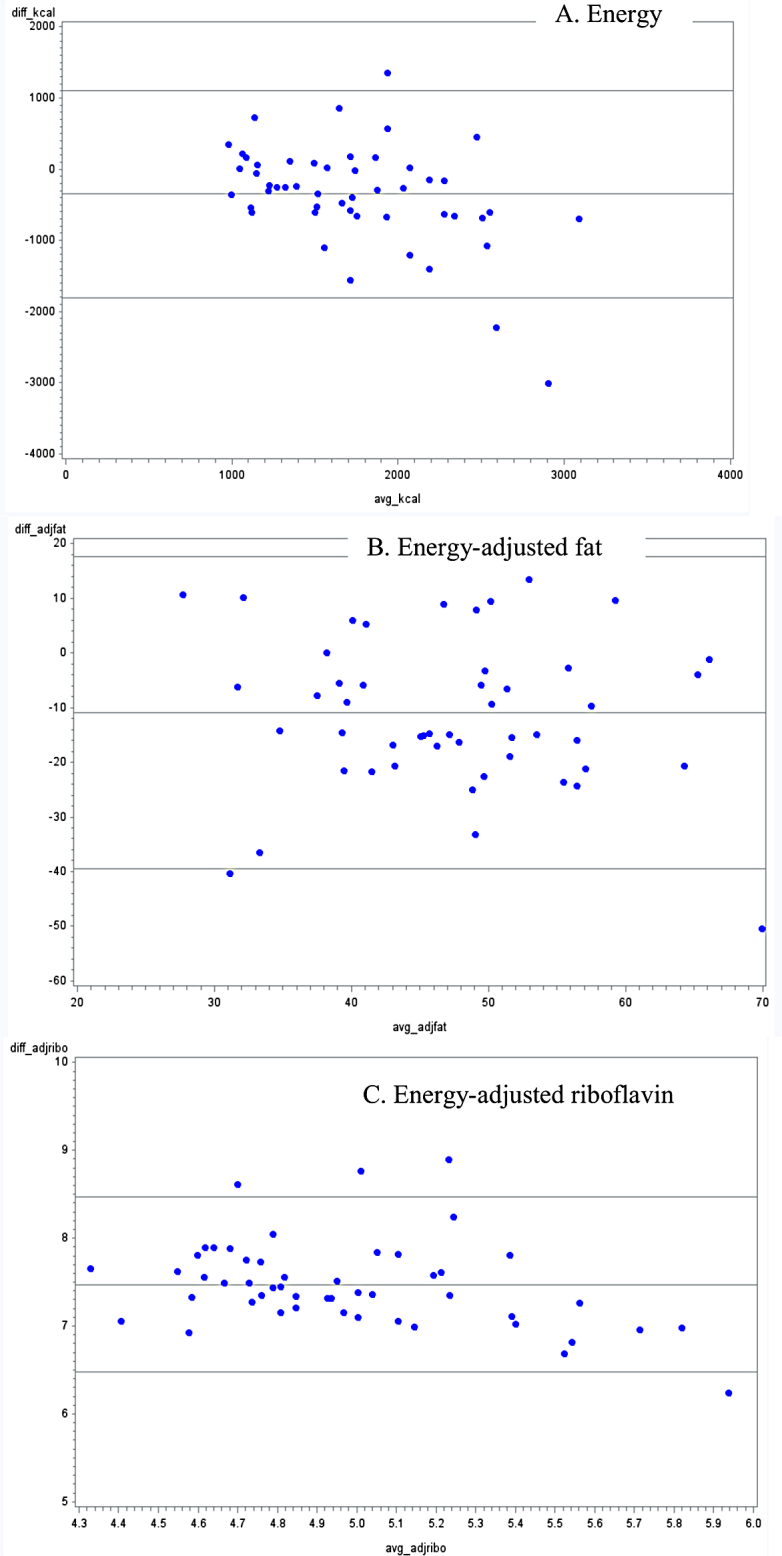
Bland-Altman plots for selected nutrients comparing a food frequency questionnaire with photos (FFQ3), with the average of three 24-hour dietary recalls in Guatemalan schoolchildren 6–11 years old.

Out of 18 nutrients, an overestimation of the unadjusted daily mean intake was found in 61% of the nutrients for FFQ1, in 89% for FFQ2 and in 94% of nutrients for FFQ3. Mean intake of sodium and cholesterol were significantly underestimated when assessed by FFQ1 (p-values <0.0001 and 0.04, respectively). For energy adjusted daily mean intake (data not shown), an overestimation was found in 55% of the nutrients for FFQ1, in 78% for FFQ2 and in 72% of nutrients for FFQ3. Bland-Altman plots also show bias in overestimation of many nutrient intakes from FFQ methods when compared with 24-hour dietary recalls, particularly for FFQ 2 and FFQ 3 (Figs 1–3, appendix; for space constraints, an example each of micronutrient, macronutrient and energy are presented). However, this bias was not consistent across all levels of intake, across all nutrients, or across all FFQs.

Neither unadjusted nor energy-adjusted Pearson correlation coefficients showed a consistent pattern of comparison for any of the individual FFQs (Tables 1–3), though they tended to be lower for energy-adjusted nutrients in FFQ2 and FFQ3. Correcting for residual measurement error increased correlations for many nutrients, particularly for FFQ1 (niacin) and FFQ3 (potassium). We classified the correlation coefficients into 3 categories: less than 0.30 (low correlation), between 0.30 and less than 0.50 (medium correlation) and 0.50 or more (high correlation) [38]. The highest adjusted Pearson correlation coefficients were found comparing FFQ2 and 24-hour dietary recalls, including 0.66 and 0.67 (for vitamins B12 and C), and 0.74 (for riboflavin). The range of adjusted coefficients obtained comparing FFQ1 and 24-hour dietary recalls was 0.07 (for protein) to 0.53 and 0.54 (for riboflavin and cholesterol). The range of adjusted coefficients obtained comparing FFQ3 and 24-hour dietary recalls was -0.09 (for sodium) to 0.51 (for vitamin B12).

The proportions of individuals classified into the same quartile by the FFQ and the mean of the three 24-hour dietary recalls varied for each FFQ, for both unadjusted and energy-adjusted intakes (Tables 4 and 5). For FFQ1, these ranged from 21.7% (magnesium) to 47.8% (vitamin B12); for FFQ2, from 26.5% (sodium) to 57.1% (vitamin B12); and for FFQ3, from 20.0% (sodium) to 54.0% (niacin). The highest percentage of participants classified into the opposite quartile of consumption (e.g., gross misclassification) was similar for all the FFQs, though the nutrient differed, with 13.0% grossly misclassified for carbohydrate consumption using FFQ1, 12.2% for niacin using FFQ2 and 12% for folic acid and sodium using FFQ3. Concordance of classification of individuals into the same or adjacent quartiles by both methods was greatest in FFQ2 for both unadjusted and energy-adjusted nutrients, with ranges from 69.4% for vitamin C (unadjusted) to 93.9% for vitamin B12 (unadjusted), and 61.2% for energy-adjusted niacin to 95.9% for energy-adjusted vitamin B12. Weighted kappa statistics for unadjusted and energy-adjusted nutrients show moderate to very good agreement between the two methods, except for fat (unadjusted) and magnesium (unadjusted) from FFQ1; energy-adjusted carbohydrate and protein from FFQ2; sodium (unadjusted) and cholesterol (energy-adjusted) from FFQ3; and vitamin C (energy-adjusted) from FFQ1, FFQ2 and FFQ3.

**Table 4:** Cross classification of unadjusted energy and nutrient intakes of Guatemalan children aged 6–11 years old, comparing three different types of FFQs and an average of three 24-hour dietary recalls.

	FFQ1				FFQ2				FFQ3			
	n = 46				n = 49				n = 50			
	Same Quartile	Same or Within 1 Quartile	Opposite Quartile	κ_w_	Same Quartile	Same or Within 1 Quartile	Opposite Quartile	κ_w_	Same Quartile	Same or Within 1 Quartile	Opposite Quartile	κ_w_
Energy (kcal)	41.3	71.7	8.7	0.60	38.8	83.7	6.1	0.59	40.0	84.0	6.0	0.61
Carbohydrate (g)	26.1	65.2	13.0	0.42	38.8	79.6	8.2	0.59	30.0	80.0	6.0	0.49
Fat (g)	26.1	69.6	6.5	0.40	32.7	73.5	8.2	0.51	44.0	84.0	2.0	0.63
Protein (g)	26.1	60.9	13.0	0.42	40.8	81.6	8.2	0.62	42.0	82.0	4.0	0.61
Iron (mg)	43.5	69.6	10.9	0.63	44.9	83.7	2.0	0.63	38.0	80.0	4.0	0.57
Calcium (mg)	34.8	78.3	10.9	0.56	34.7	71.4	8.2	0.54	34.0	78.0	4.0	0.52
Niacin (mg)	32.6	69.6	6.5	0.48	30.6	73.5	12.2	0.50	54.0	78.0	6.0	0.71
Riboflavin (mg)	45.7	84.8	4.3	0.64	42.9	89.8	0.0	0.63	42.0	92.0	2.0	0.63
Thiamin (mg)	34.8	67.4	6.5	0.52	53.1	85.7	2.0	0.71	40.0	76.0	4.0	0.56
Pyridoxine (mg)	39.1	78.3	6.5	0.58	49.0	91.8	0.0	0.66	46.0	82.0	4.0	0.65
Cholesterol (mg)	45.7	84.8	4.3	0.63	34.7	83.7	0.0	0.53	28.0	76.0	4.0	0.46
Folic acid (μg)	32.6	73.9	8.7	0.50	30.6	79.6	4.1	0.50	38.0	70.0	12.0	0.57
Vitamin B12 (μg)	47.8	76.1	4.3	0.64	57.1	93.9	4.1	0.75	40.0	92.0	2.0	0.61
Zinc (mg)	28.3	69.6	8.7	0.46	34.7	81.6	2.0	0.54	42.0	72.0	0.0	0.58
Magnesium (mg)	21.7	69.6	8.7	0.38	38.8	75.5	2.0	0.57	36.0	82.0	4.0	0.54
Potassium (mg)	30.4	69.6	4.3	0.47	42.9	79.6	4.1	0.63	40.0	80.0	2.0	0.58
Sodium (mg)	39.1	67.4	6.5	0.56	26.5	79.6	6.1	0.45	20.0	68.0	12.0	0.37
Vitamin A/RE(μg)	26.1	78.3	6.5	0.44	28.6	75.5	2.0	0.45	30.0	72.0	8.0	0.47
Vitamin C (mg)	39.1	78.3	8.7	0.59	36.7	69.4	4.1	0.53	40.0	80.0	0.0	0.56

FFQ, Food frequency questionnaire; RE, retinol equivalents; FFQ1, Food frequency questionnaire without portion sizes; FFQ2, Food frequency questionnaire with portion sizes, FFQ3, Food frequency questionnaire with pictures of median portion sizes. Values are reported as percentages.

Same quartile = exact match in ranking from both methods (FFQ vs. 24-hour dietary recall); Opposite quartile = gross misclassification of ranked individuals by each methods (FFQ vs. 24hour dietary recall). Note: The expected values by chance are 25% for the same quartile or opposite quartile and 62.5% for within one quartile.

**Table 5:** Cross classification of energy-adjusted nutrient intakes of Guatemalan children aged 6–11 years old, comparing three different types of FFQs and an average of three 24-hour dietary recalls.

	FFQ1				FFQ2				FFQ3			
	n = 46				n = 49				n = 50			
	Same Quartile	Same or Within 1 Quartile	Opposite Quartile	κ_w_	Same Quartile	Same or Within 1 Quartile	Opposite Quartile	κ_w_	Same Quartile	Same or Within 1 Quartile	Opposite Quartile	κ_w_
Carbohydrate (g)	39.1	69.6	13.0	0.58	34.7	67.3	14.3	0.40	30.0	74.0	4.0	0.46
Fat (g)	26.1	63.0	15.2	0.42	36.7	73.5	10.2	0.56	30.0	80.0	6.0	0.48
Protein (g)	30.4	63.0	15.2	0.47	20.4	65.3	14.3	0.34	38.0	76.0	8.0	0.57
Iron (mg)	39.1	71.7	10.9	0.59	38.8	81.6	2.0	0.58	40.0	72.0	12.0	0.61
Calcium (mg)	23.9	78.3	4.3	0.43	30.6	75.5	8.2	0.49	24.0	70.0	8.0	0.41
Niacin (mg)	37.0	78.3	6.5	0.55	26.5	61.2	12.2	0.41	30.0	72.0	6.0	0.46
Riboflavin (mg)	30.4	87.0	2.2	0.51	57.1	87.8	2.0	0.74	30.0	86.0	6.0	0.51
Thiamin (mg)	32.6	67.4	4.3	0.49	28.6	83.7	6.1	0.49	34.0	78.0	4.0	0.52
Pyridoxine (mg)	39.1	76.1	6.5	0.59	46.9	87.8	0.0	0.65	32.0	80.0	4.0	0.51
Cholesterol (mg)	32.6	80.4	0.0	0.51	32.7	65.3	10.2	0.50	22.0	62.0	6.0	0.34
Folic acid (μg)	32.6	78.3	6.5	0.51	36.7	67.3	2.0	0.53	30.0	66.0	6.0	0.44
Vitamin B12 (μg)	50.0	80.4	6.5	0.68	59.2	95.9	2.0	0.76	48.0	84.0	0.0	0.65
Zinc (mg)	37.0	73.9	2.2	0.54	32.7	77.6	6.1	0.52	32.0	82.0	10.0	0.52
Magnesium (mg)	39.1	71.7	10.9	0.58	38.8	77.6	6.1	0.58	42.0	78.0	10.0	0.62
Potassium (mg)	41.3	78.3	4.3	0.60	28.6	67.3	2.0	0.44	40.0	70.0	10.0	0.59
Sodium (mg)	28.3	71.7	6.5	0.45	30.6	73.5	10.2	0.50	28.0	72.0	14.0	0.45
Vitamin A/RE(μg)	37.0	80.4	8.7	0.56	34.7	79.6	8.2	0.56	24.0	76.0	10.0	0.43
Vitamin C (mg)	30.4	78.3	6.5	-0.05	40.8	83.7	4.1	-0.05	32.0	80.0	8.0	-0.08

FFQ, Food frequency questionnaire; RE, retinol equivalents; FFQ1, Food frequency questionnaire without portion sizes; FFQ2, Food frequency questionnaire with portion sizes, FFQ3, Food frequency questionnaire with pictures of median portion sizes. Values are reported as percentages.

Same quartile = exact match in ranking from both methods (FFQ vs. 24-hour dietary recall); Opposite quartile = gross misclassification of ranked individuals by each methods (FFQ vs. 24hour dietary recall). Note: The expected values by chance are 25% for the same quartile or opposite quartile and 62.5% for within one quartile.

## Discussion

To our knowledge, this is the first validation study of dietary instruments aimed at assessing intake of school aged children in semi-urban villages in Guatemala. We found that both the FFQ2 (with portion size) and FFQ3 (with photos) have moderate to good relative validity, as for most nutrients, unadjusted correlation coefficients ranged from 0.3 to 0.6. Our results follow the expected pattern, where correlation coefficients comparing an FFQ and a reference measure were higher when subjects described their own portion size (FFQ2) compared with no portion size specified (FFQ1) or portion size specified on the questionnaire (FFQ3) [39] and are similar to results from a study in Brazil, which utilized a larger sample size [40]. When comparing crude correlation coefficients in FFQ2 and FFQ3, for some nutrients, such as cholesterol and sodium, we observed higher correlations between FFQ2 and the 24-hour dietary recalls than for FFQ3. Evidence assessing the correlation coefficient of sodium between FFQs and 24-hour dietary recalls for school-age children is scarce. However, Del Pino and Friedman [40] found a similar correlation coefficient to ours (0.47 vs. 0.41, respectively) for sodium intake.

Though energy-adjustment would be assumed to improve the observed correlation between the two methods of assessment, this was not the case for some nutrients. We observed this across all three FFQs. Such an observation may indicate an over- or under-assessment of intake for such nutrients, as has been reported in previous studies [15, 17]. Our correction for measurement error helped deattenuate some of the results, though it did not do so for all nutrients across all FFQs of the study.

Overall, all FFQs overestimated energy and nutrient values, when compared with the average of three 24-hour dietary recalls. This discrepancy has been found in previous studies [40–43]. One plausible explanation may be the underlying function of FFQs: though the unit of measure received from an FFQ is in grams/ day, these questionnaires are not designed to measure absolute food consumption, but rather to rank individuals according to their intakes. For example, mean unadjusted calcium intake was statistically higher when assessed by both FFQ2 and FFQ3 compared with their corresponding 24-hour dietary recalls. Consequently, comparison between both FFQs and the 24-hour dietary recalls presented only a moderate correlation (r = 0.36 and r = 0.47, respectively). However, these results are in agreement with evidence shown by a previous systematic review [7]. Additionally of note, biases shown though Bland-Altman plots were not consistent across intake levels or across nutrients, and dependent upon the FFQ. Findings from a validity study in Brazilian children have previously shown a dependency between the difference and the average of FFQs and 24-hour recalls, leading to a greater level of error associated with extreme intake levels [44].

The FFQs were in general agreement with 24-hour dietary recalls for categorizing individuals according to energy and nutrient intake: the percentage of children classified by both methods into the same or adjacent quartiles ranged from 69.4% for vitamin C to 94% for vitamin B12. The agreement rates of children correctly classified within the same or adjacent quartile was higher than those expected by chance (i.e., 65%) for every nutrient except for protein intake assessed by FFQ1. For energy-adjusted intakes, this was the case except for niacin from FFQ2 and cholesterol from FFQ3. These results are similar and, in some cases, better than results from FFQs in other populations, but similar settings (31) and represent the utility of FFQs for ranking individuals’ intakes within a population.

We are aware of few studies that have specifically examined the validation of FFQs in Latin and Central America [17]. Dietary patterns in villages located at the outskirts of Guatemala City are different than the patterns in other countries; therefore, FFQs developed for different areas may not be applicable to these communities. To our knowledge, the only other FFQ designed for a Guatemalan population and previously validated in the country [31] was designed to measure the dietary intakes in a populations of adults in eastern Guatemala. These dietary patterns may differ from the patterns of semi-urban communities. Additionally, FFQs developed for adults but applied to children prove to grossly overestimate the child’s dietary intake [38, 45], in many cases to a larger degree than observed by the FFQs and nutrients in our study. The original FFQ developed for adults included almost half of the number of food items included in our three FFQs constructed for children, which could be an indicator of a more heterogeneous food pattern among children.

Our study highlights several of the challenges present when studying dietary intakes in children. We found varying results based on each individual FFQ under study. While both FFQ1 and FFQ3 were relatively easy to administer and less of a burden on the respondent, both showed lower correlation with the comparison method (24-hour dietary recalls) than FFQ2. However all three FFQs performed moderately well in classifying individuals based on intake categories, which is the true benefit of using an FFQ over other methods for dietary assessment. Future studies should consider these advantages and disadvantages—including, but not limited to, participant burden (the time involved, intellectual capacity for measurement, and resources available), desired outcome (e.g., categorization of individuals vs. precise measurement of intake), and setting (access to resources, knowledge of the indigenous diet)—when choosing a FFQ type for assessing dietary intakes in children. Further research is needed to validate each FFQ with objective biological measures to assess their pertinent use in nutrient assessment.

Our study presents some limitations. Data were collected over a 3 year period: July, 2006 for FFQ1, July, 2007 for FFQ2, and February, 2009 for FFQ3. The 24-hour dietary recalls were collected on the same week as the FFQs. Since the month—and, therefore, season—of data collection for FFQ1 and FFQ2 differed from that of FFQ3, seasonal effects may not have been completely accounted for. Also, the FFQs did not include weekends in their measurement; our results therefore may not fully reflect the overall usual intake of children, as it was restricted to school days.

Additionally, although many studies have assessed the relative validity of FFQs which included median portion sizes or photographs of median portions this technique does not overcome some of the inherent limitations of both FFQs and 24-hour dietary recalls, including reliance on respondent’s memory and perception of serving sizes. Also, our photograph atlas used with FFQ3 only included one portion size per food item; it has been suggested that more photograph choices (e.g., 5–8) could decrease portion size estimation error [46]. Direct observation could have been used to help eliminate error due to recall bias; however, we did not find this appropriate for this setting, due to the burden on the participant (specifically, the time and space) and resources necessary (specifically, trained individuals and hours necessary).

Finally, it is not feasible to assume that parents are valid surrogate reporters of children’s food intake. However, it has been stated that children’s recall skills before 12 years of age are limited, as are their ability to estimate and indicate portion sizes, and general knowledge of foods [47]. As found in a systematic review [48], almost all of the validity and reliability studies of dietary assessments in children younger than 9 years old included adult assistance for the child’s reported intake. Although the respondents were not the children themselves, we were able to obtain responses from the caregiver closest to the child, to provide a relatively reliable proxy.

We also acknowledge several strengths to our study including the methods used and FFQ development according to the local eating patterns. The original FFQ used as a basis to create our three FFQs had been previously tested in a similar setting among adults [31]. We were able to add to the reliability of this FFQ for measurement of nutrients within this specific population by utilizing foods commonly consumed by children in the community, to capture local eating patterns. The FFQs were designed to assess the dietary patterns of middle-low and low income individuals, and therefore may be applicable in other towns within the country. Our decision to focus on children’s consumption patterns adds to the paucity of literature on this topic. Although there is no gold standard to use as a reference for the validation of dietary assessment methods [49] we used the 24-hour dietary recall as our comparator, which is a frequently used method for validation purposes and was suitable for our population [50]. Finally, all interviews were conducted face-to-face by trained personnel and discrepancies were able to be clarified by talking directly with the respondent.

In conclusion, our FFQs show moderate relative validity for measuring selected nutrients in children 6–11 years old living within this setting, and moderate to good agreement for ranking individuals based on intake categories, when compared with results from 24-hour dietary recalls. This study suggests that these FFQs can fairly measure energy and nutrient intakes for children in a peri-urban area of Guatemala City. However, more evidence is needed to evaluate the instrument’s reproducibility and applicability in similar populations.

Word count: 4,029

## Supporting Information

S1 Dataset Data for participants receiving FFQ without portion sizes, and their corresponding 24h recalls. (SAS7BDAT)

S2 Dataset Data for participants receiving the FFQ with portion sizes, and their corresponding 24h recalls. (SAS7BDAT)

S3 Dataset Data for participants receiving the FFQ with photographs, and their corresponding 24h recalls. (SAS7BDAT)
